# Extracellular Superoxide Dismutase: Growth Promoter or Tumor Suppressor?

**DOI:** 10.1155/2016/3612589

**Published:** 2016-05-12

**Authors:** Mikko O. Laukkanen

**Affiliations:** IRCCS SDN, Via Comunale Margherita 484-538, 80131 Naples, Italy

## Abstract

Extracellular superoxide dismutase (SOD3) gene transfer to tissue damage results in increased healing, increased cell proliferation, decreased apoptosis, and decreased inflammatory cell infiltration. At molecular level,* in vivo* SOD3 overexpression reduces superoxide anion (O_2_^−^) concentration and increases mitogen kinase activation suggesting that SOD3 could have life-supporting characteristics. The hypothesis is further strengthened by the observations showing significantly increased mortality in conditional knockout mice. However, in cancer SOD3 has been shown to either increase or decrease cell proliferation and survival depending on the model system used, indicating that SOD3-derived growth mechanisms are not completely understood. In this paper, the author reviews the main discoveries in SOD3-dependent growth regulation and signal transduction.

## 1. Introduction

Extracellular superoxide dismutase (EC-SOD, SOD3) [1, 2], similar to cytosolic CuZn-SOD (SOD1) [3] and mitochondrial MnSOD (SOD2) [4, 5], catalyzes the dismutation of superoxide anion (O_2_^−^) into hydrogen peroxide (H_2_O_2_) (in this review reactive oxygen species refer to O_2_^−^ and H_2_O_2_), which to date is the only reported physiological function of the enzyme. Thus, the cellular effects of SOD enzyme activity are caused by changes in the local concentrations of O_2_^−^ and H_2_O_2_, which are second messengers in signal transduction that have an impact on growth capacity and the transformation of primary cells. Although the enzymes have a significant therapeutic potential their delivery to injury site is challenging due to limitations in gene transfer efficiency. Hence researchers have developed SOD mimics that function similarly with SOD enzymes regulating redox balance with consequent impact on growth, differentiation, and death [6–10]. The importance of local regulation of reactive oxygen species (ROS) by SOD3 has been highlighted by our previous studies of local and systemic delivery of* sod3* via adenovirus to sites of cardiovascular injury: both gene transfer methods increase plasma SOD activity, but only the local gene delivery demonstrates a therapeutic response [11]. The data is supported by observations reporting that Arg-213-Gly mutation at C-terminal end of SOD3 reduces the affinity of the enzyme to heparan sulphate proteoglycans of endothelial cells thus increasing plasma SOD3 concentration by 10-fold [12, 13]. The mice carrying Arg-213-Gly mutation have tissue level changes, such as increased neutrophil mediated inflammation, cellular degeneration and premature aging, abnormal gait, and reduced lifetime that may be result of increased neutrophil ROS production [14]. Based on the abovementioned data decreased SOD3 content at cell membranes impairs life-supporting cellular functions. Notably, H_2_O_2_ can have toxic effects on cellular functions at high concentrations, thus suggesting a need to regulate ROS production in the tissue environment. Indeed, a number of reports have demonstrated tight regulation of SOD3 expression at the transcriptional, posttranscriptional, and posttranslational levels [12, 15–23]. This regulation is influenced by various factors, most importantly by the level of O_2_^−^ substrate and the reaction end product H_2_O_2_ [23–25].

## 2. Therapeutic Effects of SOD3 Overexpression

**Figure 1:**
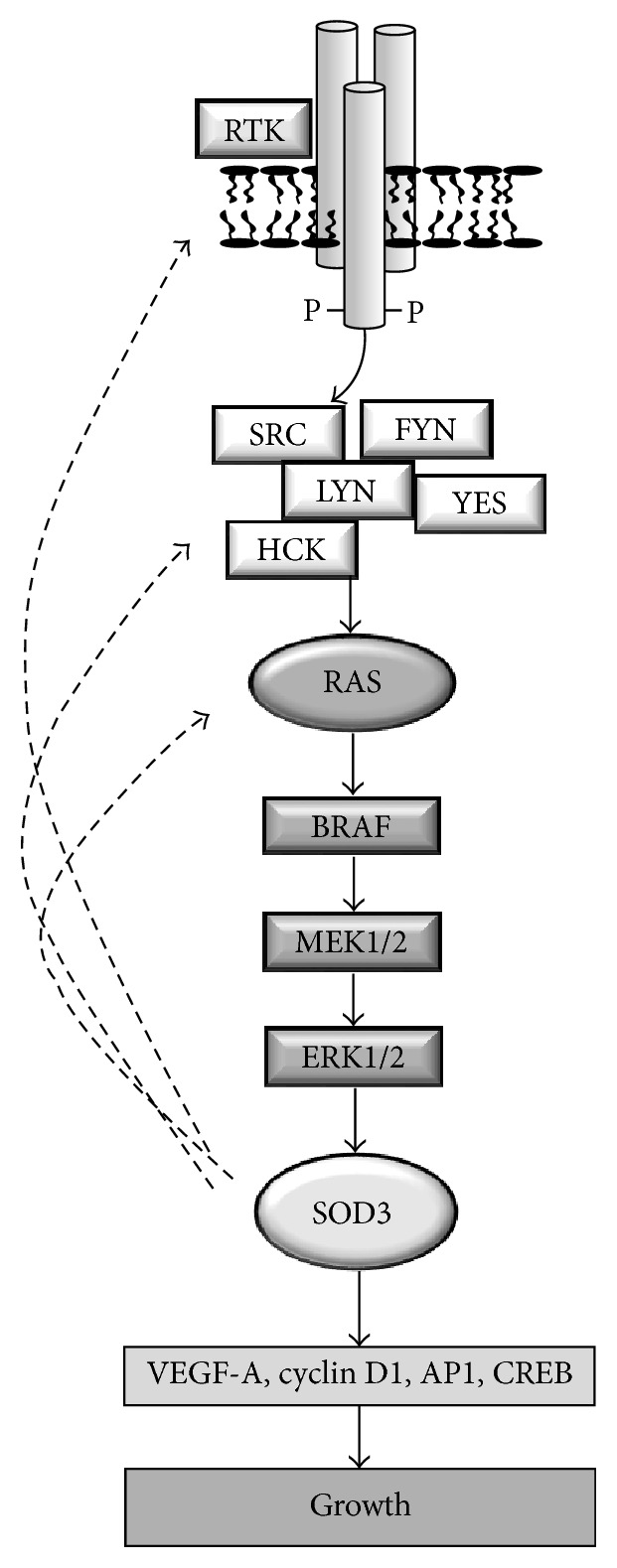
Suggested positive feedback loop in SOD3 signal transduction. Phosphorylation of RTKs activates the cell membrane associated SRC proto-oncogene family members that contribute to RAS GTP loading and stimulation of mitogenic signal transduction to BRAF, MEK1/2, and ERK1/2 kinases.* In vitro* transient transfection of* RAS*,* BRAF*,* MEK1/2*, and* ERK1/2* increases both* SOD3* mRNA and protein expression hence suggesting mitogen pathway induced SOD3 synthesis. SOD3 production results in increased synthesis of growth promoters, such as VEGF and cyclin D1, and increased activation of activator protein 1 (AP1) and cAMP response element-binding protein (CREB). Importantly, SOD3 activates cell surface receptor tyrosine kinases (RTKs), increases phosphorylation of SRC family members, and regulates the GTP loading to small GTPases, such as RAS.

One of the first milestones in SOD3 research was the discovery of the tissue-protective nature of the enzyme in cardiovascular models. The earliest observations reported reduced cardiovascular damage by recombinant SOD3 administration [26–30]; these observations were confirmed by a series of gene transfer studies [11, 24, 31–39] and later reviewed in [40–44]. Characteristically, treatment of cardiovascular tissues with SOD3 reduces the extent of the damage, increases the healing process, improves cardiac function, reduces the remodeling of vasculature, attenuates apoptosis, inhibits inflammatory and smooth muscle cell migration, and increases cell proliferation and endothelial cell layer recovery. The role of SOD3 in neoangiogenesis is less clear. We have reported increased endothelialization and reduced macrophage and smooth muscle cell migration with consequent long-term inhibition of neointima formation in rabbit denudation and in rabbit in-stent models [11, 38], suggesting a role for the enzyme in vascular cell proliferation and inflammatory cell migration. We have further demonstrated, using rat hind limb injury model, SOD3-dependent increases in tissue injury recovery that were mediated by activation of mitogen signal transduction with consequent increased satellite cell proliferation in muscles [24]; by activation of antiapoptotic signaling that involved increased extracellular signal regulated kinase 1/2 (ERK1/2), protein kinase B (AKT), and forkhead box O3a (FOXO3a) activation [39]; and by reduction of macrophage-specific inflammation, which was correlated with reduced expression of the inflammatory cytokines tumor necrosis factor *α* (TNF*α*), interleukin 1*α* (IL1*α*), interleukin 6 (IL6), macrophage inflammatory protein 2 (MIP2), and monocyte chemotactic protein 1 (MCP-1) and the adhesion molecules vascular adhesion molecule (VCAM), intercellular adhesion molecule (ICAM), P-selectin, and E-selectin [36]. Although we did not observe increased neoangiogenesis by overexpressing SOD3, another recent study performed in SOD3 knockout mice suggested defective vessel formation in the absence of the enzyme. The authors demonstrated that SOD3 does not directly promote vascular endothelial growth factor receptor (VEGFR) activation but it is able to enhance the ability of VEGF ligand to phosphorylate VEGF-R [45]. Thus,* in vivo* data suggest that SOD3 expression activates growth-promoting, antiapoptotic, and anti-inflammatory signal transduction pathways in cardiovascular models (Figure 1).

The function of SOD3 in lung models has been investigated using SOD3 null and transgenic mice. The earliest observations suggested that SOD3 null mice had a significantly shortened life span and experienced death associated with lung edema under conditions of hyperoxia [46]. These observations were confirmed in conditional knockout mice that showed reduced survival associated with disorders resembling adult respiratory distress syndrome, such as thickening of alveolar septa, increased inflammation, hemorrhage, and loss of patent alveoli [47]. Hence, the lung model data support results obtained from cardiovascular damage models, suggesting survival-supporting and growth-promoting roles for SOD3 in the tissue environment.

The most dramatic prosurvival effect of SOD3 has been observed in total body irradiation (TBI) studies. In one study, intravenous administration of 0.5 × 10^6^ mesenchymal stem cells (MSCs) previously transduced with SOD3-expressing adenovirus multiplicity of infection (MOI) 2000 resulted in a 90% survival rate 35 days after 9 Gy TBI without hematopoietic stem cell (HSC) transfusion, whereas 90% of control animals died [48]. These data were confirmed in a study of mice receiving 5.81 Gy TBI, which showed a similar survival rate [49]. In this study, in the absence of HSC transplantation, the transfusion of MSCs (1 × 10^6^) transduced with SOD3-expressing adenovirus (MOI 50) resulted in a 90% 30-day survival rate compared to a 20% survival rate in control animals. Blood value analysis 10 days after TBI demonstrated that there were eightfold higher white blood cell counts (1.1 × 10^8^ in controls versus 8.9 × 10^8^ in the SOD3 group), 40-fold higher platelets values (2.4 × 10^9^ in controls versus 97 × 10^9^ in the SOD3 group), and significantly increased hemoglobin levels (105 g/L in controls versus 128 g/L in the SOD3 group) in SOD3-treated animals compared to controls [49]. Although the authors concluded that the increased survival was caused by significantly decreased apoptosis in SOD3-treated animals, another possible survival mechanism could be increased cycling of primitive HSCs with consequent hematopoietic cell differentiation. The data provided by Gan and coworkers suggested that the gene expression of members (i.e.,* p53*,* p21*, and* p16*) of the* p53*-mediated growth arrest pathway was reduced in MSC-SOD3-transplanted animals [49], supporting the hypothesis that increased SOD3-driven mitogen stimulus in the bone marrow together with reduced apoptosis might explain the increased survival after TBI observed in SOD3-treated animals. Previous studies have suggested a common bone marrow niche and homing site for HSCs and MSCs [50, 51], thus indicating that SOD3-treated MSCs could have a paracrine effect on quiescent HSCs, inducing them to proliferate and to differentiate by directly affecting primitive progenitor cell cycling or via erythropoietin signaling [52]. Hence, the* in vivo* data observed from various animal models suggest that SOD3 maintains normal tissue homeostasis by promoting cell survival and proliferation.

## 3. Hydrogen Peroxide Action in Signal Transduction

Hydrogen peroxide regulates a number of cellular functions, such as cell proliferation, differentiation, migration, and survival. The first evidence that H_2_O_2_ could function as a second messenger came from studies demonstrating increased H_2_O_2_ production in association with increased platelet-derived growth factor (PDGF), epithelial growth factor (EGF), and vascular endothelial growth factor (VEGF) receptor tyrosine kinase phosphorylation with simultaneously reduced protein tyrosine phosphatase (PTP) activity [53–55]. In general, ROS are able to affect cell signaling by two mechanisms: (1) by inactivating PTPs, thereby increasing tyrosine kinase phosphorylation, and (2) by directly oxidizing tyrosine kinase receptors, causing their phosphorylation [56, 57].

H_2_O_2_ and O_2_^∙−^ are known to be involved in the initiation of tumorigenesis and in malignant transformation [58]. In addition to increasing cell proliferation, survival, and migration, H_2_O_2_ activates SRC family proto-oncogenes, which regulate vascular development and vascular permeability [59], the latter of which is an early step in tumor stroma development [60]. The role of H_2_O_2_-derived signaling in the later phase of tumor development allows cancer cell survival in hypoxic environments by maintaining activation of the AKT pathway, with a consequent increased expression of hypoxia inducible factor 1*α* [61].

## 4. SOD3 Expression in Tumorigenesis

Previous data have suggested that there is a correlation between increased* sod3* mRNA production and increased growth of benign tumors [62], indicating a role for SOD3 in early tumorigenesis.* In vitro* studies have supported this conclusion by demonstrating that moderate overexpression of SOD3 stimulates mouse primary embryonic fibroblast (MEF) cell proliferation, mimicking the RAS oncogene response in primary cells [63] and further corroborating the close relationship of SOD3 expression and cellular growth. Consistent with these results,* sod3* mRNA synthesis is upregulated at low RAS activation levels; however,* sod3* mRNA expression, which negatively correlates with* mir21* expression, is strongly downregulated when the RAS activation level increases to ≥10-fold relative to parental cells [23]. In contrast to the case in benign growth, SOD3 expression is progressively downregulated in a number of cancers and cancer cell lines [62, 64–67], correlating with the RAS activation level [23], which suggests that* sod3* could be a prognostic differentiation marker. Silencing of the* SOD3* gene can be divided into reversible immediate events and stable late events. Immediate events following RAS activation occur via SOD3 self-regulation through small RAS GTPase regulatory genes,* mir21* upregulation, and p38 MAPK phosphorylation [23, 68–72], whereas late regulatory events consist of DNA methylation and histone acetylation [15, 16, 72–74]. The correlation of decreased* SOD3* expression with increased malignancy has led to the hypothesis that SOD3 could function as a tumor suppressor that must be silenced to allow the progression of carcinogenesis [66]. Although the hypothesis is feasible based on conventional tumor suppressor gene silencing mechanisms, the mechanisms of how reduced* SOD3* expression could increase transformed cell proliferation have not been fully elucidated.

## 5. SOD3 as a Growth Promoter in Tumorigenesis

As mentioned above, SOD3 has been shown to promote normal primary cell proliferation in various model systems [11, 24, 33, 34, 38, 63]. The close connection of SOD3 to growth-associated signal transduction was demonstrated in a recent microarray functional KEGG and GO pathway analysis suggesting that the highest number of SOD3-affected genes was in the MAPK signaling (254 genes, *p* < 0.02) and endothelial cell proliferation pathways (33 genes, *p* < 0.018). Other significantly affected pathways included various cancer-associated signal transduction and cell proliferation pathways [75]. We have previously shown that RAS-BRAF-MEK1/2-ERK1/2, a major signal transduction pathway in cancer, activates* SOD3* mRNA expression and enzyme activity* in vitro* and* in vivo*, which then increases GTP loading to RAS [24]. These data suggest the existence of a positive feedback loop that maintains the mitogen pathway in a phosphorylated state, inducing growth-supporting and antiapoptotic signal transduction pathways in injured tissues [24, 39] (Figure 1). We have further shown that thyroid stimulating hormone (TSH), cAMP-PKA, and PLC-Ca^2+^ increase* sod3* production in thyroid cells, demonstrating that SOD3 in the thyroid contributes to cell proliferation and differentiation [62]. The role of SOD3 in growth promotion was further strengthened by data indicating that expression of SOD3 induces the activation of AP-1, c-Jun, and CRE promoter regions; increased expression of FOXO3a and FOXQ1 transcription factors; and increased expression of the cell cycle proteins cyclin D1, cell division cycle 25A (CDC25A), and proliferating cell nuclear antigen (PCNA) [24, 39, 63]. Importantly, H_2_O_2_ treatment of cells has been shown to stimulate* SOD3* mRNA synthesis and mimic SOD3 function in cells that were treated with N-acetylcysteine (NAC) and diphenyleneiodonium (DPI), suggesting substrate specific regulation of the enzyme [23, 24, 75].

**Figure 2:**
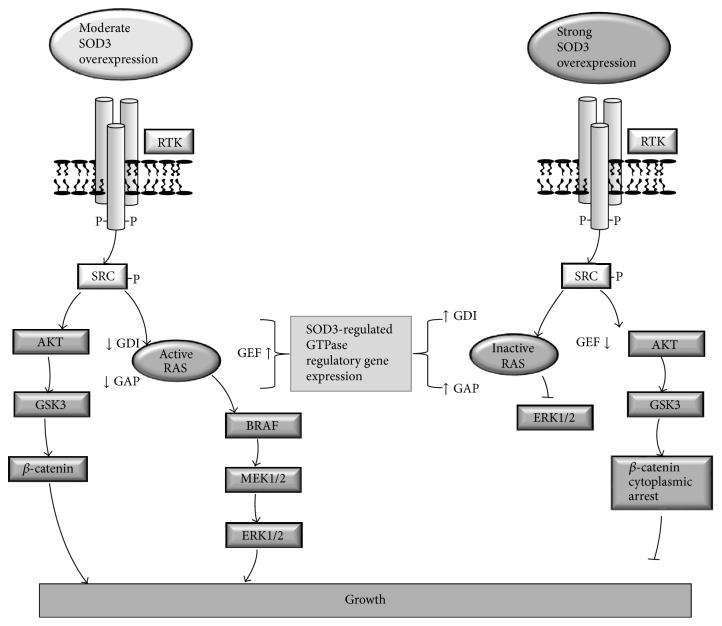
Suggested model for dose-dependent effect of SOD3 on RAS activation and *β*-catenin cellular localization. Moderately increased SOD3 expression at cell membranes promotes cell membrane bound RAS GTP loading by activating GEF expression and by inhibiting GAP and GDI synthesis causing increased RAS-ERK1/2 signaling. Robustly increased SOD3 expression inhibits RAS GTP loading by inhibiting GEF expression and by activating GAP and GDI synthesis causing decreased RAS-ERK1/2 signaling. Moderately increased SOD3 expression promotes AKT and GSK3 phosphorylation and *β*-catenin nuclear entry, whereas robustly increased SOD3 expression arrests *β*-catenin to cytoplasm by increasing the expression of* WWTR1*,* SNAI2*, and* AXIN2*. Note that both moderate and robust SOD3 expressions increase the phosphorylation of RTKs, SRCs, AKT, and GSK3. SOD3 dose-dependent signal transduction regulation occurs at the level of small GTPases and *β*-catenin cytoplasm-nuclear localization.

Signal transduction studies have suggested that cell membrane bound SOD3 increases the phosphorylation of the cell membrane tyrosine kinase (RTK) receptors, epidermal growth factor receptor (EGFR), erb-b2 receptor tyrosine kinase 2 (ERBB2), receptor-like tyrosine kinase (RYK), anaplastic lymphoma kinase (ALK), Fms-like tyrosine kinase 3 (FLT3), Ephrin A10 (EPHA10), and VEGF-R [45, 67, 75]; cell membrane-associated signaling molecules, such as the SRC proto-oncogene family members HCK, FYN, SRC, YES, and LYN; and cytoplasmic signaling molecules, including AKT, glycogen synthase kinase 3 (GSK3), and *β*-catenin [75]. Hence, SOD3 overexpression activates two main growth-related signal transduction pathways, RAS-ERK1/2 and *β*-catenin cascades (Figure 2).

Interestingly, recent studies have indicated that reducing the expression of SOD3 to physiological levels can increase the growth of transformed malignant cells* in vitro* and* in vivo*. Based on* in vivo* data, VEGF-C-driven SOD3 expression increases the tumorigenesis and metastasis of xenografted mammary cells [67]. Additionally, knockdown of VEGF-C in mammary cancer cell lines significantly reduces the expression of SOD3, tumor formation, and metastasis of the cells, whereas restoration of SOD3 expression in VEGF-C knockdown cells to the levels of control cells with carcinogenic characteristics partly recovers the aggressiveness of the cells, increasing both primary tumor growth and metastasis [67]. The growth-promoting effects of SOD3 are further supported by studies in cancer cell lines harboring decreased endogenous* SOD3* expression; transfection of* SOD3* into these cell lines results in* in vitro* and* in vivo* growth selection of cells, favoring those with modestly increased* SOD3* levels.* In vitro* transfection of high* SOD3* concentrations into cancer cells followed by mixed population long-term culture results in apoptosis of cells with high supraphysiological concentrations of* SOD3* plasmid, whereas cells that contained moderately increased* SOD3* expression compared to control cancer cells took over the culture due to their increased proliferation capacity.* In vivo* studies with xenografted* luciferase*-marked cells support this observation, showing an initial decrease in tumorigenesis and in luciferase signal in tumors derived from SOD3-transfected cancer cells containing a strong increase in* SOD3* mRNA expression. The initial decreased growth phase was followed by faster tumor development and* in vivo* selection of cells, which contained moderately increased* SOD3* mRNA expression levels compared to control cell-derived tumors [63]. Thus, SOD3, by affecting local ROS concentrations, might have progrowth characteristics in early tumorigenesis as a mediator of the RAS oncogene and, in certain cellular environments, may work in coordination with other growth factors that stimulate cancer cell proliferation.

## 6. SOD3 as a Growth Suppressor in Cancer

Various studies have demonstrated cancer growth suppression caused by supraphysiological* SOD3* overexpression* in vitro* and* in vivo*. In general, these studies have been performed using cells transfected with* SOD3* at high concentrations or using cells transduced with adenovirus expressing* SOD3* [63, 75–79], which induces strong mRNA production, thus reaching supraphysiological SOD3 and H_2_O_2_ levels for three to four days [80]. High expression of* SOD3* results in DNA damage and activation of the DNA damage response (DDR), including phosphorylation of histone H2AX, phosphorylation of checkpoint kinase1/2 (CHK1/2), phosphorylation of p53, increased production of p21, and consequent growth arrest and apoptosis [63]. Additionally, supraphysiological SOD3 expression in anaplastic cancer cells activates AKT-GSK3-*β*-catenin signaling but prevents *β*-catenin nuclear transfer by increasing the gene expression of WW domain-containing transcription regulator protein 1 (*WWTR1*), snail homolog 2 (*SNAI2*), and axis inhibition protein 2 (*AXIN2*) [75], which are responsible for *β*-catenin cytoplasmic arrest, binding, and degradation, respectively [81–83].

At the tissue level, supraphysiological SOD3 overexpression correlates with reduced oxidative stress marker 4-hydroxynonenal staining in xenografted tumors and decreased intracellular dihydroethidium staining in cancer cells transduced with adenovirus expressing* SOD3* [77, 78]. Functionally, supraphysiological overexpression of SOD3 inhibits the nuclear localization of NF-*κ*B, reduces VEGF-A expression, decreases cell proliferation, inhibits tumor growth, decreases metastasis (suggesting a reduction of* in vivo* cancer cell migration), and increases apoptosis [76–78]. Furthermore, SOD3 has been shown to affect hypoxia inducible factor HIF-1*α* signaling, which enables vascular growth, thus regulating tumor progression. supraphysiological* SOD3* overexpression by adenovirus (MOI 50–100) decreases HIF-1*α* levels by inducing degradation, whereas virus doses of MOI 25 or less have minor or no impact on HIF-1*α* levels [84].

## 7. SOD3 Affects Growth in a Dose-Dependent Manner

*SOD3* overexpression studies have demonstrated a dual role for the enzyme in growth regulation depending on the expression level of the enzyme: rescued or moderately increased SOD3 expression promotes cell proliferation, whereas supraphysiological overexpression of the enzyme causes growth arrest and apoptosis [63, 67, 75, 77, 78]. Notably, moderately increased SOD3 levels stimulate cell proliferation, mimicking the function of the RAS oncogene in primary cultures and causing mitogenic burst followed by growth arrest-related senescence, immortalization of primary cells, and even transformation of the cells together with additional changes in cellular signaling [63, 85–87]. Interestingly, our data have suggested the existence of SOD3 dose-dependent regulation of downstream signal transduction at the level of RAS small GTPases [24, 75]. A moderate twofold increase in SOD3 activity in tissues markedly increases RAS GTP loading and downstream growth signaling [24, 75], whereas robust supraphysiological SOD3 overexpression decreases RAS, RAC, RHO, and CDC42 activation by regulating the gene expression of regulators of these small GTPases. Mechanistically, moderate SOD3 expression increases the mRNA expression of guanine nucleotide exchange factors (GEFs) responsible for GTP loading to GTPases and decreases the expression of GTPase activating proteins (GAPs) and guanine nucleotide dissociation inhibitors, which are responsible for maintaining GDP-RAS association and inhibiting localization of GTPases to cell membranes, where they are activated [88–90]. Robust supraphysiological expression of SOD3, which has been shown to reduce primary tumor growth metastasis and cancer cell proliferation, reduces the expression of GEFs and increases the expression of GAPs and GDIs, resulting in inhibition of the activation of downstream ERK1/2 kinases [75] (Figure 2). Hence, modification of the gene expression of regulators of these small GTPases is a cornerstone in SOD3-derived control of the RAS-ERK1/2 mitogen pathway activation and cellular growth. It is important to note that, in line with the effect of SOD3 on cellular functions, the effect of H_2_O_2_ is concentration dependent. Low physiological (<0.7 *μ*M) concentration induces growth, whereas concentrations above 50 *μ*M induce DNA damage and senescence [91]. Therefore, SOD3-driven signal transduction resembles H_2_O_2_-activated signaling.

## 8. Conclusions

The role of SOD3 in tumorigenesis is only partly solved. Recent studies have suggested that SOD3 has dose-dependent effects on primary tumor growth and metastasis activity that, however, may depend on the ability of different kinds of tumor cells to detoxify ROS differently. Thus, several avenues of research must still be pursued. Most importantly, the* in vitro* results that showed that moderate SOD3 mRNA overexpression induces primary cell immortalization and transformation should be repeated using* in vivo* model systems, and the mechanism of this effect should be further elucidated. Second, increased* SOD3* mRNA expression correlates with increased benign growth and decreased* SOD3* mRNA expression correlates with increased malignant progression, thus creating a dilemma regarding the signal transduction differences between primary and transformed cells. The presented hypothesis suggesting increased aggressiveness of cancer cells caused by decreased SOD3 mRNA expression requires a mechanistic explanation. Furthermore, as strong expression of* SOD3* mRNA induces apoptosis and death of cancer cells, it would be of great interest to determine if* SOD3* gene regulation in certain cellular conditions allows supraphysiological expression of the enzyme, causing cellular death, thus suggesting tumor suppressor characteristics for SOD3. Although the function of SOD3 as a regulator of cellular growth has been well established by a number of studies, the enzyme itself might not be a suitable cancer drug or druggable target molecule. Rather, SOD3-related signal transduction studies might indicate mediators of tumor progression, which could then be useful targets for preclinical and clinical studies.
